# Effect of heart rate on hemodynamics in patient-specific intracranial aneurysms: An in vitro study

**DOI:** 10.1371/journal.pone.0359941

**Published:** 2026-10-29

**Authors:** Baha Al-Deen T. El-Khader, Nikhil Shirdade, Jana Korte, Daniel Behme, Philipp Berg, Ephraim W. Church, Melissa C. Brindise

**Affiliations:** 1 Department of Mechanical Engineering, The Pennsylvania State University, University Park, Pennsylvania, United States of America; 2 Department of Fluid dynamics and Technical flows, University of Magdeburg, Magdeburg, Germany; 3 STIMULATE Research Campus, University of Magdeburg, Magdeburg, Germany; 4 Clinic for Neuroradiology, University of Magdeburg, Magdeburg, Germany; 5 Chair in Healthcare Telematics and Medical Engineering, University of Magdeburg, Magdeburg, Germany; 6 Department of Neurosurgery, Penn State Health, Hershey, Pennsylvania, United States of America; University of Southern California, UNITED STATES OF AMERICA

## Abstract

The rupture of an intracranial aneurysm (IA) has been linked to local hemodynamic environments, but it remains unclear how transient physiological states such as elevated heart rate modulate those environments, and whether different aneurysm geometries respond similarly. Here, we used volumetric particle tracking velocimetry (PTV) to measure 3D velocity fields in two patient-specific IA phantoms, a right middle cerebral artery (MCA) aneurysm and a left vertebral artery (LVA) aneurysm, under three heart rates representative of rest (60 bpm), active movement (100 bpm), and exercise (137 bpm). From these measurements, we quantified coherent structures, turbulent kinetic energy (TKE), wall shear stress (WSS), and oscillatory shear index (OSI). Increasing heart rate did not simply increase peak inflow; it altered the temporal organization of the flow. In the MCA aneurysm, higher heart rates produced a stronger, more anchored intra-saccular vortex and elevated coherent-structure strength and TKE that persisted into mid- and end-diastole, along with broader spatial coverage of elevated WSS that remained high beyond peak systole. The LVA aneurysm exhibited a more muted response, with minimal changes in streamline topology and WSS across heart rates. These results show that heart rate can drive prolonged high-shear, high-energy loading within the cardiac cycle, but that this amplification is morphology-dependent and non-monotonic rather than universal.

## 1. Introduction

Unruptured intracranial aneurysms (IAs) affect approximately 3% of the global population [1] and 1–6% of adults in the United States [2,3]. Ruptured IAs cause hemorrhagic strokes, resulting in mortality rates of 40–50% and morbidity rates of 10–20% [4,5]. Surgical intervention is possible for aneurysms at high rupture risk but carries an approximately 7% complication risks, making it unsuitable for stable IAs, which represent roughly two-thirds of cases. Accurate rupture risk assessment remains challenging, as the mechanisms underlying IA pathology are not fully understood.

Previous studies identified aneurysm geometry, clinical factors (e.g., gender, smoking, hypertension), and hemodynamics as key contributors to intracranial aneurysm (IA) growth and rupture [6]. Specifically, abnormal hemodynamics play a primary role by inducing adaptive endothelial cell responses to pathological mechanical stresses [7]. Meng et al. proposed that high wall shear stress (WSS) with positive gradients initiates growth and rupture in smaller IAs, whereas low WSS with elevated oscillatory shear index (OSI) promotes enlargement in larger IAs, reflecting complex aneurysm pathophysiology [8]. High turbulent kinetic energy (TKE) has also been associated with IA rupture [9]. Additionally, flow instabilities leading to significant spatio-temporal WSS gradients, complex flow patterns, concentrated jets, and localized impingement points have been correlated with IA growth and rupture [10–13].

Intense physical exertion and emotional stress, characterized by rapid changes in heart rate and blood pressure, have been linked to IA rupture [14]. These observations suggest that these physiological factors may precipitate adverse hemodynamic conditions within IAs, but exactly how remains unclear. Studies exploring heart rate effects on IA flow dynamics consistently indicate that the Womersley number, proportional to heart rate, influences vortex propagation within aneurysms. Lower Womersley numbers allow vortices to travel deeper into the IA sac during acceleration and retreat during deceleration [15,16]. However, relationships between heart rate and hemodynamic parameters like WSS remain inconsistent. Jiang and Strother observed increased WSS with rising heart rate [14], whereas Miah et al. and Venugopal et al. found no significant change [17,18]. Bowker et al. reported mixed results depending on geometry [7]. Collectively, these findings highlight the need for studies that evaluate heart rate-based effects in the context of other risk factors to clarify its conditional role in IA hemodynamics.

In this study, we evaluate how heart rate interacts with aneurysm morphology and inflow jet orientation to shape the intra-aneurysmal hemodynamic environment. Specifically, we test whether physiologically realistic changes in heart rate, spanning resting, moderate, and elevated states, alter not only the intensity of the flow but also the organization, persistence, and wall loading of intra-saccular structures. Unlike prior studies that have largely reported bulk or peak hemodynamic metrics under varied heart rates, we focus on how heart rate reshapes the spatio-temporal structure of vortices, turbulent kinetic energy, and wall shear patterns within patient-specific geometries. In doing so, we directly address the inconsistent WSS–heart-rate relationships in the literature by examining them in the context of aneurysm morphology and inflow configuration. To do this, we manufactured phantom models of two patient-specific intracranial aneurysms: a right middle cerebral artery (R-MCA) aneurysm and a left vertebral artery (LVA) aneurysm. Volumetric particle tracking velocimetry (PTV) was used to resolve the 3D velocity field in each aneurysm at three heart rates (60, 100, and 137 beats per minute, bpm), and derived hemodynamic metrics including coherent structures, TKE, WSS, OSI were computed and compared. This approach allows us to identify geometry-dependent responses to heart rate, distinguish amplifying versus buffering behavior across aneurysm types, and assess how heart rate shifts not just peak stresses but their spatial footprint and duration within the cardiac cycle.

## 2. Methods

### 2.1. Aneurysm geometries

For the R-MCA aneurysm, magnetic resonance angiography (MRA) scan images were captured at Penn State’s Milton S. Hershey Medical Center (Hershey, PA, USA). The data were retrospectively obtained for this study on May 5, 2022; the Pennsylvania State University Institutional Review Board approved this study which was exempt from informed consent. A 3D model of the R-MCA aneurysm (Fig 1a) was created by extruding the MRA images. The aneurysm was separated from the vasculature manually, using SolidWorks 2022 (Dassault Systèmes, Boston, USA). The LVA aneurysm (Fig 1b) was captured with 3D digital subtraction angiography (DSA), the images were segmentated in MeVisLab v3.2. (MeVis Medical Solutions AG, Bremen, Germany) via region growing. The aneurysm was separated from the vasculature manually through observation using MeshMixer 3.5 (Autodesk, Inc, CA, USA). For both flow phantoms, the inlet and outlet vessels were extended so that the models could be connected into the flow loop. A polyvinyl alcohol (PVA) phantom model was 3D-printed (TAZ, LULZBOT) using STL files of the aneurysms. The PVA models were individually embedded into acrylic boxes designed for each geometry. Polydimethylsiloxane (PDMS) Sylgard 184 (refractive index n=1.413) was poured in the acrylic box around the PVA model and left to cure for 48 hours. After curing, the printed PVA model was dissolved with water in a sonicator. Both aneurysm phantoms were fabricated as block-cast PDMS models rather than thin-walled compliant vascular models. Consequently, there was no discrete vascular wall thickness; instead, the vascular lumen was surrounded by a relatively large bulk volume of PDMS that was constrained by the acrylic housing. The models were therefore treated as effectively rigid during the pulsatile-flow experiments, and pressure-induced wall deformation was not considered an intended degree of freedom in the measurements.

**Fig 1:**
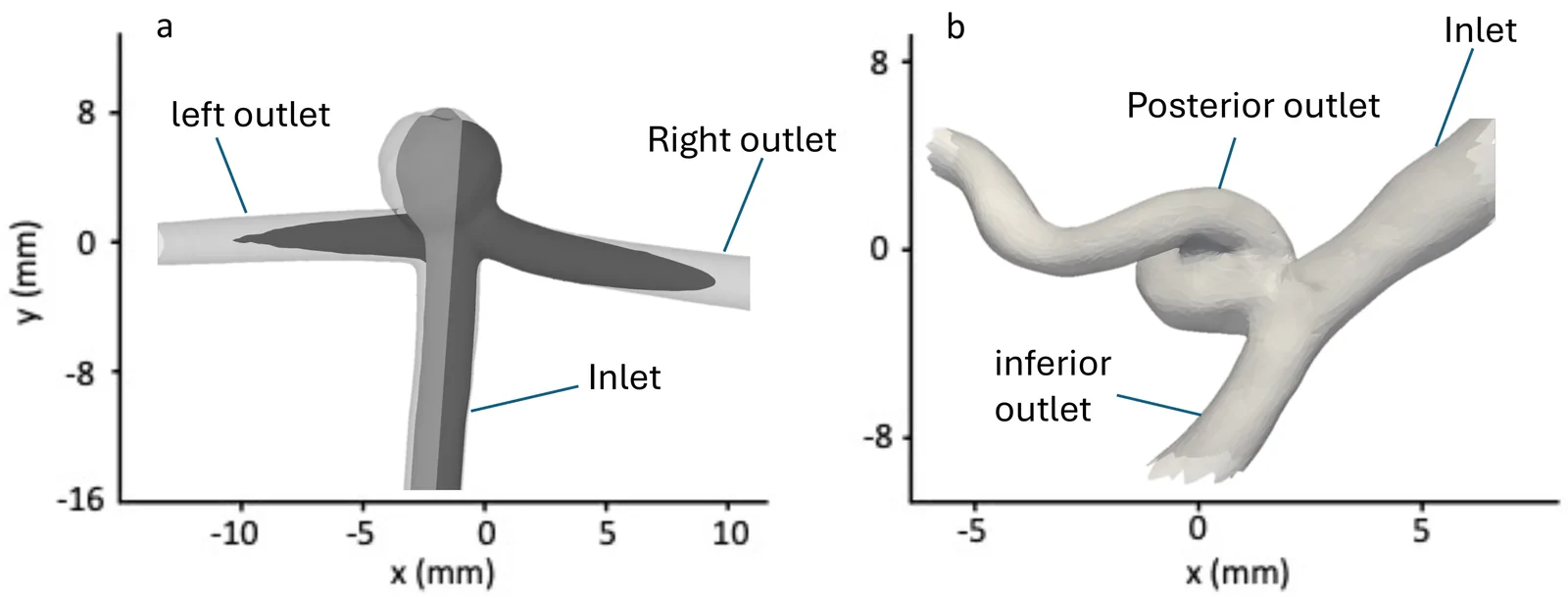
In vitro segmented (a) MCA and (b) LVA models.

### 2.2. Physiological flow loop

The flow loop and volumetric PTV setup used for this study was the same as in our prior study (El-Khader et al., 2026); we refer the reader to this paper for a detailed schematic of the experimental setup [19]. A computer-controlled gear pump (Ismatec, Glattbrugg, Switzerland) was used to generate the pulsatile inflow waveforms. To monitor the generated flow properties, pressure transducers (Omega, PX309) were placed before the inlet and after the outlets of the IAs. Ultrasonic flowmeters (Transonic Inc.) were placed upstream and downstream of the IAs. The measured pressure waveforms for the 60, 100, and 137 bpm conditions are shown in Fig 2. The corresponding mean, maximum, minimum, and pulse pressures are summarized in Table 1. No additional valves, prescribed outlet-resistance elements, or downstream compliance elements were included in the flow loop. Individual branch outlet flow rates were neither prescribed nor measured separately. Consequently, flow partition among the outlets developed passively according to the hydraulic resistance of the patient-specific geometry and downstream tubing. The downstream experimental configuration was maintained unchanged across the three heart-rate conditions for each model. There was at least 150-diameters length of tubing before the inlet of each IA to ensure a fully developed flow profile. The working fluid was a blood-analog solution composed of 44.1 wt% water, 34.5 wt% glycerol, and 21.4 wt% urea and was refractive-index matched to the PDMS model (n = 1.431). The working-fluid refractive index was measured and confirmed using a refractometer. This composition has been shown to reproduce relevant blood-analog properties; the measured fluid density was 1142 kg/m³ and the kinematic viscosity was 3.47 × 10^−6^ m²/s [20]. Each model was additionally submerged in a surrounding index-matching bath composed of 44.4 vol% water and 55.6 vol% glycerol (n = 1.413) to minimize optical distortion at the external PDMS interface. The refractive index of the surrounding fluid was likewise measured and confirmed using a refractometer. For this study, three physiologically motivated heart-rate conditions were investigated: 60, 100, and 137 bpm, representing resting, moderate-activity, and elevated-heart-rate conditions, respectively. The experiments were designed to reproduce changes in inflow associated with increasing heart rate rather than to vary cardiac frequency at a fixed flow rate. Accordingly, both the waveform frequency and inflow amplitude were varied between conditions. The mean flow rates were approximately 98, 131, and 158 mL/min, with corresponding peak flow rates of approximately 150, 202, and 239 mL/min for the 60, 100, and 137 bpm conditions, respectively. Peak inflow amplitudes at 100 and 137 bpm were approximately 1.3 and 1.6 times that of the 60 bpm condition. The imposed inflow waveforms are shown in Fig 3, and the corresponding geometric characteristics and dimensional/dimensionless flow parameters are summarized in Table 2. Reynolds and Womersley numbers were calculated as: ${Re}_{m}=u_{m}D/v$, and Womersley number $Wo=\frac{D}{2}\sqrt{2\pi f/v}$, respectively, where $u_{m}$ is the average velocity at the inlet, D is the inlet diameter, v is the measured kinematic viscosity of the blood-analog fluid, and f is cardiac frequency. The same inflow waveform was imposed on the MCA and LVA models at each heart-rate condition to enable a controlled comparison of their geometric response to identical inflow forcing. Although the imposed waveform was physiologically motivated for the MCA, it was intentionally retained for the LVA rather than replaced with a territory-specific vertebral artery waveform, as doing so would introduce an additional inflow condition variable into the cross-model comparison. The inlet diameters of the MCA and LVA models were similar (3.45 and 3.50 mm, respectively), resulting in closely matched Reynolds and Womersley numbers between the two models at each heart rate, as summarized in Table 2.

**Table 1:** Mean, minimum, and maximum pressures measured upstream of the MCA and LVA at 60 bpm, 100 bpm, and 137 bpm cases.

Heart rate (bpm)	Mean pressure (mmHg)	Minimum Pressure (mmHg)	Maximum Pressure (mmHg)
**60**	40.67	20.40	73.53
**100**	60.27	16.27	104.79
**137**	70.19	−13.22	147.85

**Table 2:** Geometric characteristics and dimensional/dimensionless inlet flow parameters for the MCA and LVA models at each heart rate condition.

Model	HR (bpm)	Inlet diameter (mm)	Right/ inferioroutlet (mm)	Left/ posterioroutlet (mm)	Sac diameter (mm)	Mean flow rate (mL/min)	Peak flow rate (mL/min)	Mean Re	Peak Re	Wo
**MCA**	**60**	**3.45**	**3.00**	**2.6**	**6.72**	**97.6**	**150.3**	**173**	**266**	**2.32**
**MCA**	**100**					**130.7**	**201.5**	**232**	**357**	**3.00**
**MCA**	**137**					**158.0**	**239.2**	**280**	**424**	**3.51**
**LVA**	**60**	**3.50**	**2.75**	**1.8**	**4.86**	**97.6**	**150.3**	**171**	**263**	**2.35**
**LVA**	**100**					**130.7**	**201.5**	**228**	**352**	**3.04**
**LVA**	**137**					**158.0**	**239.2**	**276**	**418**	**3.56**

**Fig 2:**
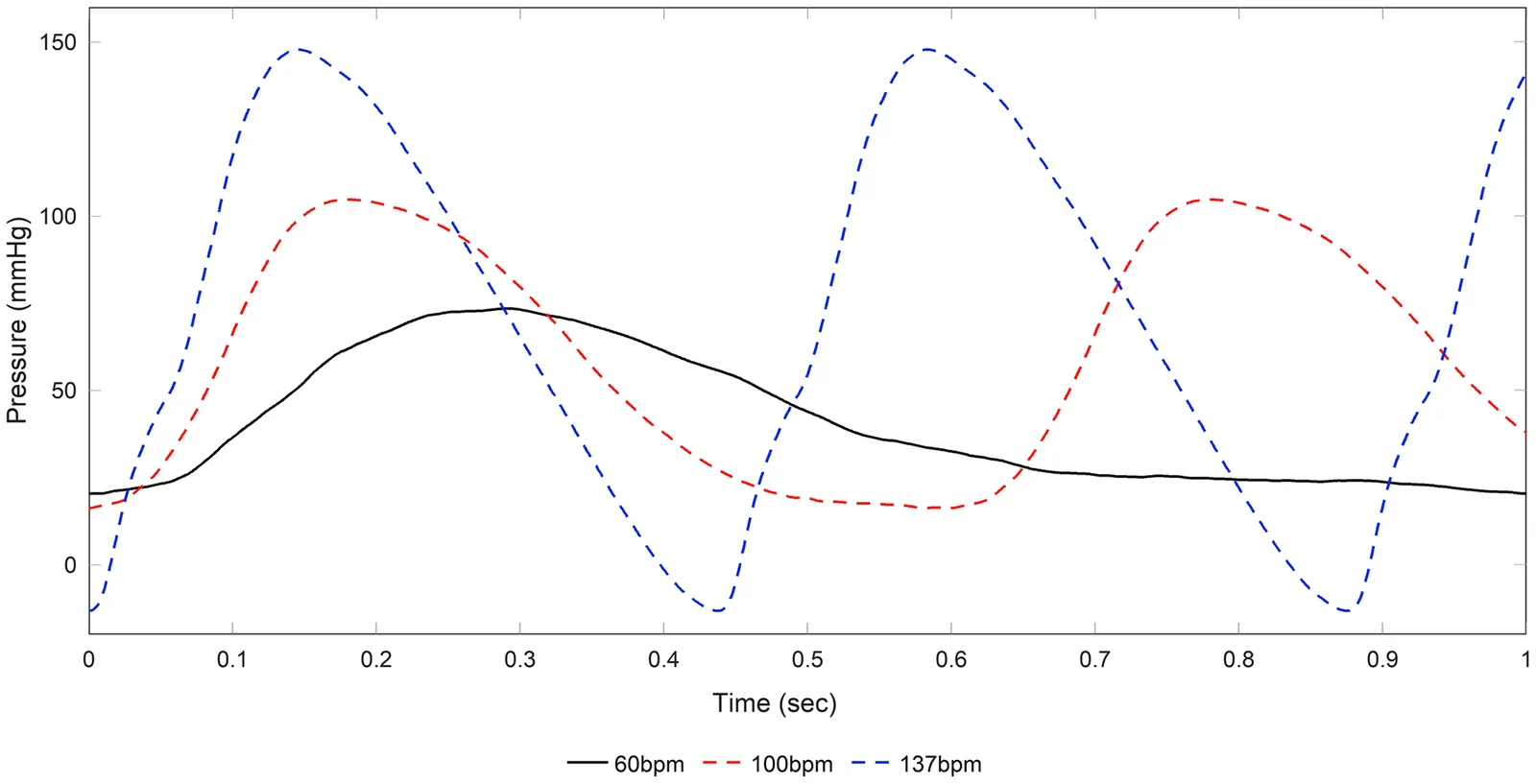
Pressure waveforms for the 60 bpm, 100 bpm, and 137 bpm cases.

**Fig 3:**
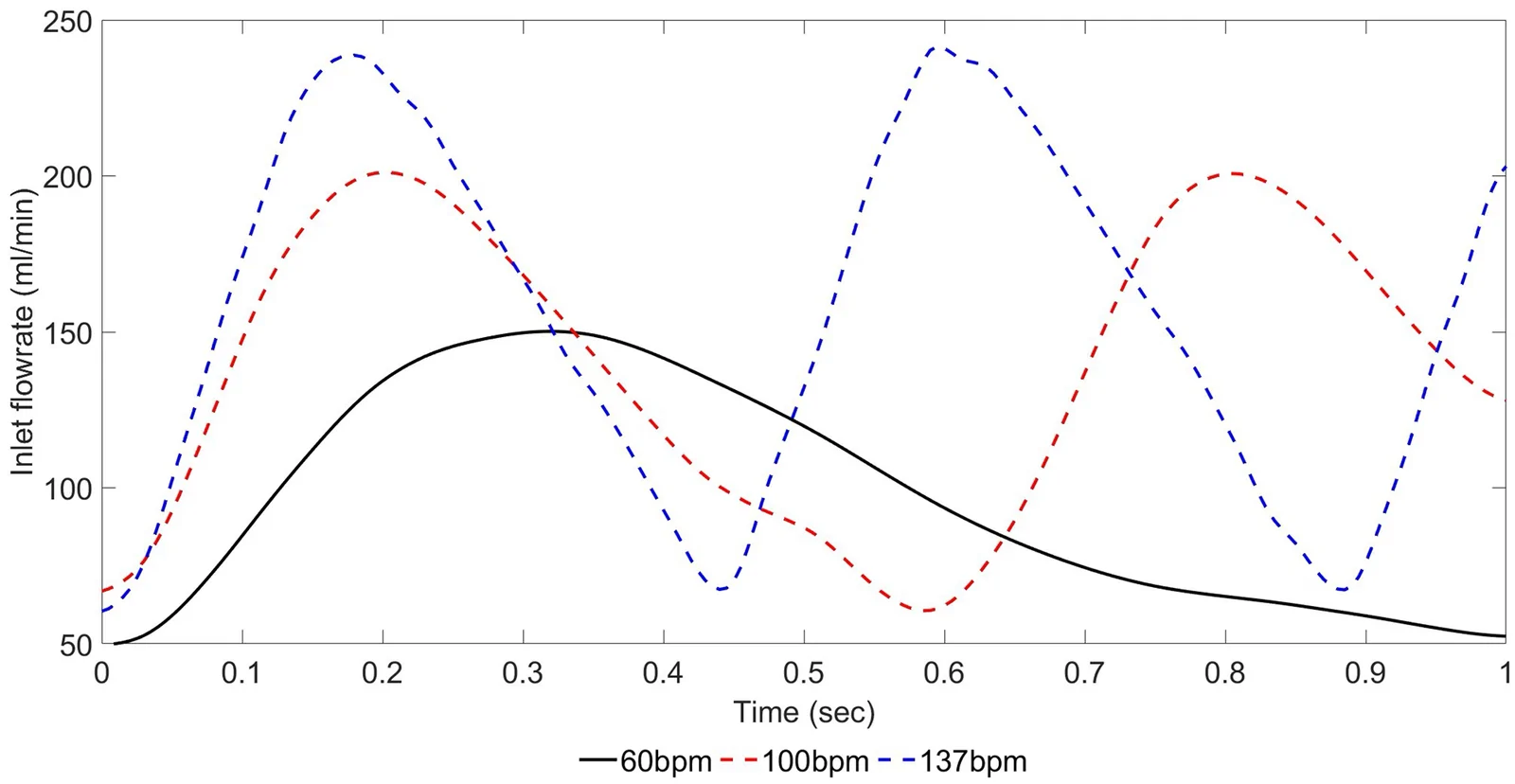
Inflow waveforms for the 60 bpm, 100 bpm, and 137 bpm cases.

### 2.3. PTV measurement technique

The working fluid was seeded with 19 µm fluorescent particles. An Nd-YLF laser (Photonics Industries, λ = 527nm) was employed to excite the fluorescent particles and four high-speed cameras (Phantom VEO440) were used to capture particle images. An optical long-pass filter was mounted on each camera to filter out the laser light wavelength. To ensure each camera observed a different angle of the measurement volume, the center camera was perpendicular to the front plane of the aneurysm, while the other cameras were angled approximately 30º to the front plane. Particle images for the R-MCA aneurysm were captured at frame rates of 1600 Hz, 2000 Hz, and 2500 Hz for the 60 bpm, 100 bpm, and 137 bpm cases, respectively. Meanwhile particle images for the LVA aneurysm were captured at frame rate frequencies of 1400 Hz (60 bpm), 1800 Hz (100 bpm), and 2200 Hz (137 bpm). The image resolution for the R-MCA aneurysm was 1280 x 852 pixels and 1792 x 952 pixels for the LVA aneurysm. These parameters were set to allow a maximum particle displacement between frames of 18 pixels and a magnification of approximately 18.4 µm/pixel. Due to the cameras’ hardware limitation and aforementioned considerations regarding maximum particle displacement, the number of pulsatile cycles captured for the R-MCA aneurysm was 13, 18, and 20 for the 60 bpm, 100 bpm, and 137 bpm cases, respectively. The number of cycles captured for the LVA aneurysm was 10 (60 bpm), 13 (100 bpm), and 14 (137 bpm).

Particle images were processed using Davis 10.0 (LaVision Inc.). Time-resolved particle images were captured and three-dimensional particle tracks were calculated using the Shake-the-box (STB) method [21] which has been shown to be less susceptible to ghost particles as well as more computationally efficient relative to tomographic particle image velocimetry (TOMO-PIV) [22]. STB processing results in a Lagrangian velocity field in the form of particle tracks. Briefly, STB uses the velocity of a particle in the current time step to predict its location in the next step. This prediction is optimized by ‘shaking’ the particle’s predicted position to minimize the residual between the predicted particle image and actual particle image for the next time step. For STB processing in this study, the allowed triangulation error was set to 3.0 voxels. The outer loop (adding particles) was set to 12 iterations and inner loop (refining particle position) was set to 8 iterations. Particle position shaking was set to 0.8 voxels and particles closer than 0.1 voxels were removed. A perspective calibration was performed in DaVis 10.0 using a LaVision calibration target (target No. 0518) and a polynomial camera-mapping model. The calibration target was traversed through nine planes across the measurement volume, with a spacing of 2.56 mm between adjacent planes, corresponding to a total calibration depth of 20.48 mm between the first and last target positions. Images of the target were acquired simultaneously by all four cameras at each plane and used to establish the three-dimensional mapping between physical and image coordinates. The initial calibration resulted in an average calibration error of 0.49 pixels. An iterative volume self-calibration procedure [23] using pre-processed particle images was subsequently performed to correct residual camera disparities, reducing the final calibration error to 0.00605 pixels.

STB tracks were first masked using an STL geometry acquired from a micro-computed tomography (μCT) scan of manufactured *in vitro* R-MCA and LVA aneurysm models. The use of the μCT scan for this step ensured the velocity masks accounted for any manufacturing errors. The velocity fields were then gridded to convert from a Lagrangian to an Eulerian framework resulting in a spatial isotropic velocity resolution of 0.3 mm^3^. A temporal grouping of the STB velocity tracks was used (4:1 ratio) for the gridding. This increased the number of particles included in the gridding process within each voxel to minimize uncertainties intrinsic to the performed averaging but consequently decreased the temporal resolution for the R-MCA aneurysm to 400 Hz, 500 Hz, and 625 Hz for the 60 bpm, 100 bpm and 137 bpm cases, respectively. The temporal resolution for the LVA aneurysm became 350 Hz, 450 Hz, and 550 Hz for the 60 bpm, 100 bpm, and 137 bpm cases, respectively. To denoise the gridded velocity fields, proper orthogonal decomposition (POD) [24] with the autonomous entropy line fit (ELF) thresholding criterion [25] was used to generate a reduced-order model of the velocity that removes noise from the velocity fields. A single pass of universal outlier detection (UOD) with a window size of 5x5x5 and a tolerance threshold of 2.5 was then used. Following this, phase averaging of the resultant pulsatile velocity fields was performed. For the R-MCA aneurysm, 13 cycles were used for phase averaging (despite capturing 18 and 20 cycles for the 100 and 137 bpm case, respectively) and for the LVA aneurysm, 10 cycles were used for phase averaging (despite capturing 13 and 14 cycles for the 100 and 137 bpm case, respectively) to ensure consistent smoothing across different heart rate cases. These masked, gridded, denoised, and phase-averaged STB velocity fields were used for all subsequent post-processing and are referred to as ‘velocity fields’ for the remainder of the paper.

### 2.4. Post-processing

Coherent structures were identified using the swirling strength (λ_ci_ criterion) [26], which evaluates the complex eigenvalues of the local velocity gradient tensor at each voxel in the measured velocity field and isolates regions of rotational motion. The magnitude of λ_ci_ was taken as a measure of vortex strength. The resulting λ_ci_ fields were used to quantify both the spatial extent and temporal persistence of coherent structures across heart rates.

To compute TKE, the Reynolds decomposition was needed to calculate the fluctuating velocity. The Reynolds decomposition can be expressed as:


$$\begin{array}{c} u_{i}^{\prime }(t)=U_{i}(t)-\left\langle U_{i}(t)\right\rangle , \end{array}$$ (1)

where $u_{i}^{\prime }(t)$ is the fluctuating velocity, $U_{i}(t)$ is the measured velocity, $\langle U_{i}(trangle$ is the mean velocity, and t is time. The mean velocity was computed using the discrete wavelet transform (DWT) as detailed in [27]. Subsequently, TKE was calculated according to:


$$\begin{array}{c} k(t)=\frac{\text{1}}{\text{2}}\left({u^{\prime }(t)}^{\text{2}}+{v^{\prime }(t)}^{\text{2}}+{w^{\prime }(t)}^{\text{2}}\right), \end{array}$$ (2)

where k(t) is the TKE, and $u^{\prime }(t),v^{\prime }(t),w^{\prime }(t)$ are the fluctuating velocities in x, y, and z directions, respectively.

Wall shear stress was calculated following the previous work of Stalder et al. [28]. All velocity gradients were computed using thin-plate spline radial basis functions [29]. WSS calculation in the near wall region used gradients beyond the biased near-wall region to reduce spatial variation in these regions, similar to the method used in previous works [11].

Time averaged WSS (TAWSS) was computed by the following equation:


$$\begin{array}{c} TAWSS=\frac{\text{1}}{T}\int_{\text{0}}^{T}|\tau_{i}|dt, \end{array}$$ (3)

where $\tau_{i}$ is the WSS vector and T is the duration of the pulsatile cycle. OSI is a dimensionless metric used to quantify the directional changes of shear stress over time. It describes the oscillatory nature of WSS on the endothelial lining of blood vessels. OSI was subsequently computed according to:


$$\begin{array}{c} OSI=\frac{\text{1}}{\text{2}}\left(\text{1}-\frac{\frac{\text{1}}{T}|\int_{\text{0}}^{T}\tau_{i}dt|}{\frac{\text{1}}{T}\int_{\text{0}}^{T}|\tau_{i}|dt}\right), \end{array}$$ (4)

Various time averaged metrics (TAM) (i.e., velocity, vorticity, TKE) were also calculated as follows:


$$\begin{array}{c} \text{TAM }=\frac{\sum_{i=\text{1}}^{N}M(t_{i})}{N}, \end{array}$$ (5)

where N is the number of time steps and M (t_i_) is the instantaneous value of some metric at the i-th time step. Additionally, the interquartile range (IQR) was calculated accordingly:


$$\begin{array}{c} \text{IQR}={\text{Q}}_{\text{3}}-{\text{Q}}_{\text{1}}, \end{array}$$ (6)

where Q_3_ is the third quartile (75^th^ percentile) of the data and Q_1_ is the first quartile (25^th^ percentile) of the data.

We note that the experiment and volumetric PTV measurements were repeated twice on different days for all cases to reduce uncertainty due to measurement or post-processing errors. Also, measured inflow velocity was verified against the upstream flowmeter reading as the reference measurement.

## 3. Results

### 3.1. Effect of heart rate on velocity fields and flow structures

Fig 4 shows 3D velocity streamlines normalized by inlet velocity for the MCA aneurysm at all heart rates and multiple time instances through the cardiac cycle. The average normalized velocity inside the aneurysm sac at peak systole was 0.077 for the 60 bpm case, and increased by 92% to 0.148 for the 100 bpm case and 110% to 0.161 for the 137 bpm case. For all heart rates, a vortex inside the IA sac with clockwise rotation was observed and its penetration depth was greatest at peak systole. However, at 60 bpm the vortex strength was elevated only at peak systole and mid diastole, whereas for the 100 bpm and 137 bpm cases it remained elevated except at mid systole, indicating insufficient decay time at the higher heart rates. Additionally, through the pulsatile cycle, the vortex-center migrated (i.e., traveled through the sac) most significantly at 60 bpm, whereas at 100 and 137 bpm the center had already returned near its start-systole position by mid-diastole. In contrast, the LVA aneurysm (S1 Fig) showed minimal differences in flow structure across heart rates. The average normalized velocity inside the IA sac was 0.172, 0.171, and 0.176, for the 60 bpm, 100 bpm, and 137 bpm cases, respectively. Further, for this geometry, vortex penetration in the IA sac was only slightly deeper at 100/137 bpm than 60 bpm, and minimal differences in jet stability and vortex migration through the pulsatile cycle were observed across cases. Collectively, these trends indicate that for flow structures, the MCA was relatively heart rate sensitive, whereas LVA showed minimal differences.

**Fig 4:**
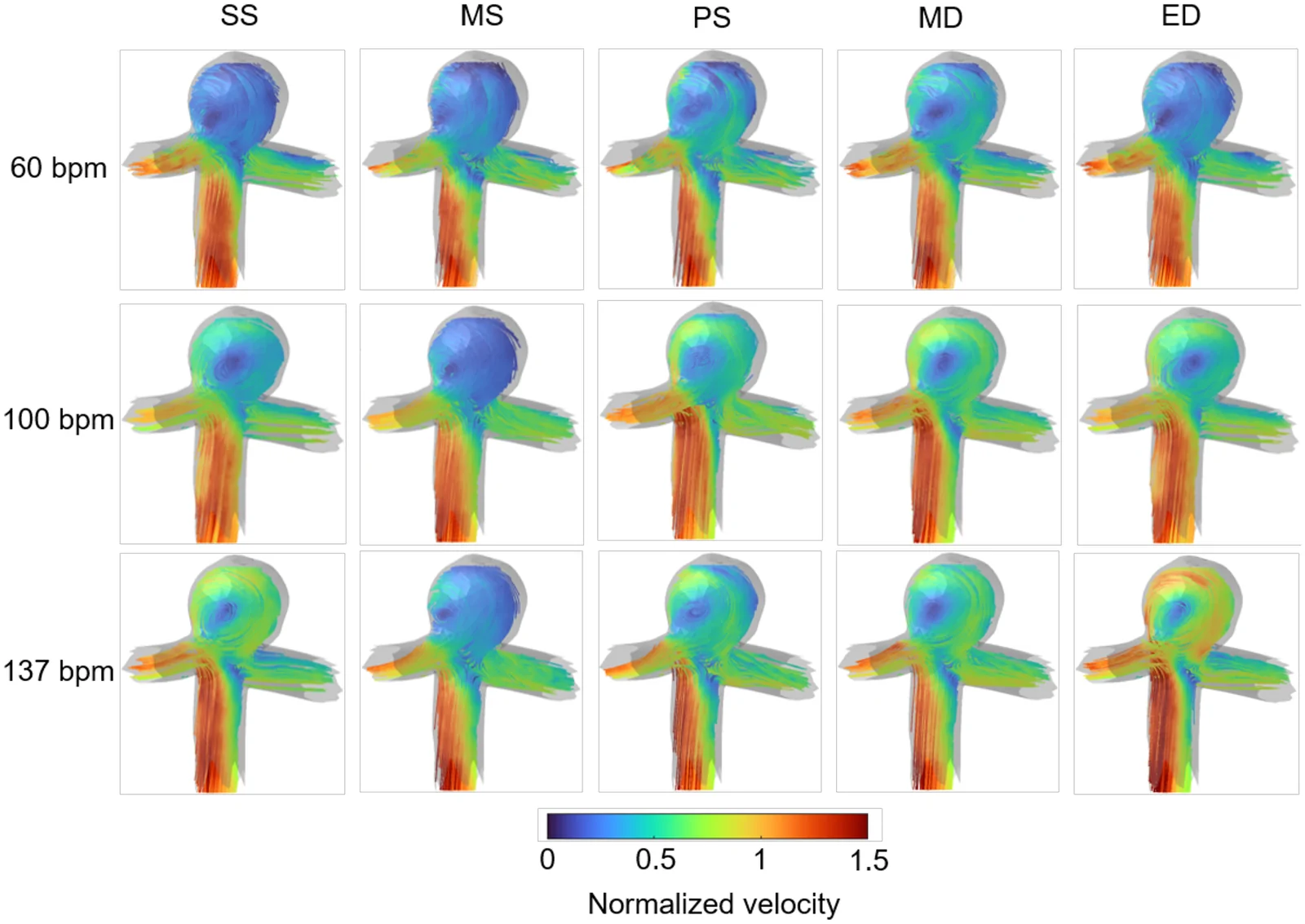
3D velocity streamlines normalized by inlet velocity for the MCA at start systole (SS), mid systole (MS), peak systole (PS), mid diastole (MD), end diastole (ED) for the 60 bpm, 100 bpm, 137 bpm cases.

Fig 5 and 6 show normalized coherent structures identified using swirling strength λ_ci_ for the MCA and LVA aneurysms, respectively, through the cardiac cycle and across heart rates. Coherent structures were thresholded by a fixed percentile (85^th^ to 99^th^) across all cases and normalized by $\frac{U_{inlet}}{{Diameter}_{inlet}}$ of each corresponding case. For both geometries, the coherent structures reflect the sac vortex wrapping around the aneurysmal sac, as previously noted in the velocity streamlines. In the MCA aneurysm, coherent structures scaled strongly with heart rate. The 60 bpm case contained the weakest and most limited regions of high-swirl, while at 100 bpm and 137 bpm, structures became stronger, more spatially extensive, and increasingly connected, occupying a large volume within the sac. For both the MCA and LVA aneurysms, maximal λ_ci_ values occurred at peak systole, but their decay through diastole differed. Specifically, in the MCA aneurysm, structures largely decay by mid-diastole at 60 bpm, persist to end-diastole at 100 bpm, and linger into both end diastole and the subsequent start systole at 137 bpm. This indicates reduced time for structural relaxation at higher heart rates. LVA exhibits the same monotonic trend with heart rate but with lower intensity and shorter lifetime, structures at 60 bpm typically vanish by mid diastole (lingering slightly longer than MCA at this heart rate), at 100 bpm persist to end diastole, and at 137 bpm extend through mid diastole and end diastole but generally do not reappear at the next start systole. Average coherent structure magnitude at peak systole/mid diastole/end diastole for the MCA aneurysm were 77.63/54.38/45.29 (60 bpm), 153.38/79.29/53.09 (100 bpm), and 128.09/82.79/76.70 (137 bpm). For the LVA aneurysm, average coherent structures were 123.91/93.82/84.06, 137.40/106.17/86.49, and 143.8/138.53/99.46 for the 60 bpm, 100 bpm, and 137 bpm cases, respectively. Thus, the formation and proliferation of coherent structures were affected by heart rate similarly for both aneurysm geometries.

**Fig 5:**
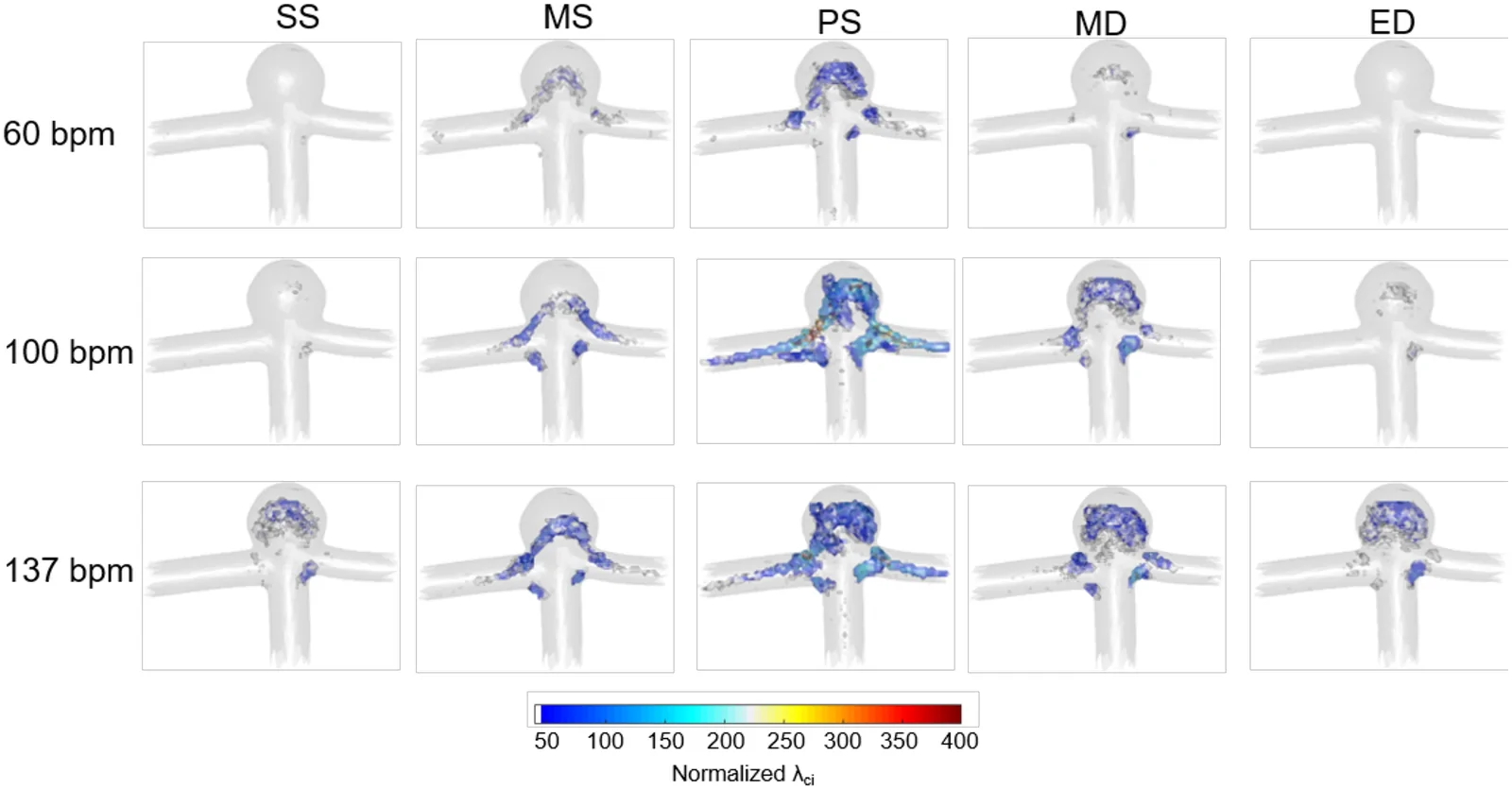
3D normalized coherent structures (λci) for the MCA at start systole (SS), mid systole (MS), peak systole (PS), mid diastole (MD), end diastole (ED) for the 60 bpm, 100 bpm, and 137 bpm cases.

**Fig 6:**
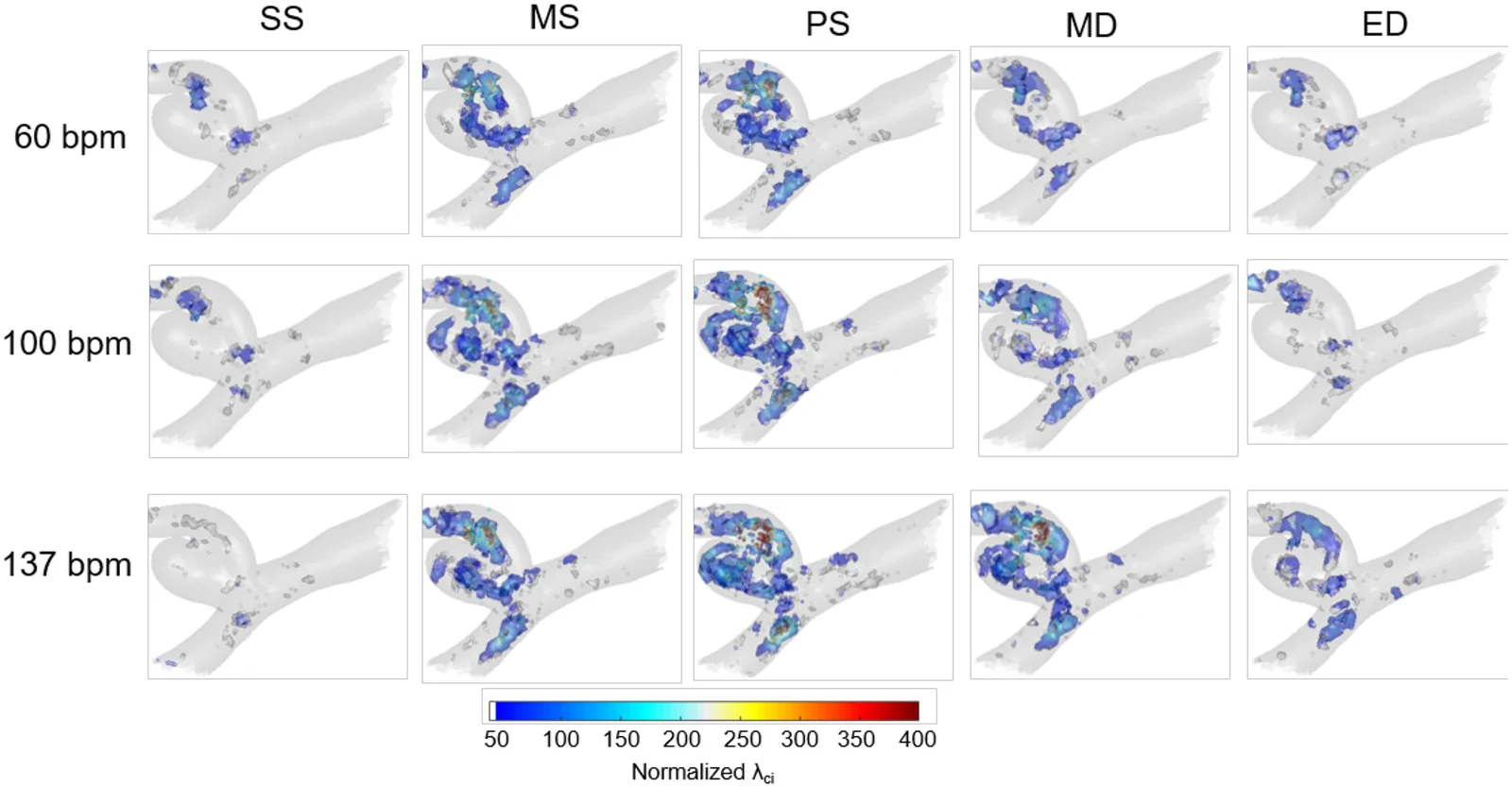
3D normalized coherent structures (λci) for the LVA at start systole (SS), mid systole (MS), peak systole (PS), mid diastole (MD), end diastole (ED) for the 60 bpm, 100 bpm, and 137 bpm cases.

### 3.2. Effect of heart rate on hemodynamic parameters: TKE, WSS, and OSI

Fig 7 and 8 show the TKE fields for the MCA and LVA aneurysms across the cardiac cycle and heart rates cases, TKE was thresholded by a fixed percentile (85^th^ to 99^th^) across all cases. In the MCA aneurysm, TKE was minimal at 60 bpm and confined to the bifurcation region with slight extension into the right outlet. TKE moderately increases at 100 bpm, extending toward the sac–right-outlet junction and slightly into the sac. At 137 bpm, TKE escalated sharply and broadened to cover the bifurcation area, both sac–outlet junctions, and inside the sac. The LVA aneurysm followed a similar monotonic trend with heart rate. At 60 bpm, TKE appeared in small pockets within the sac, bifurcation area, and outlets; at 100 bpm, these pockets enlarged and maintained higher TKE; at 137 bpm, TKE values significantly increased and high-TKE zones covered the bifurcation area, both outlets, and deep into the IA sac. Temporally, peak systole contained the largest TKE. For the 60 bpm and 100 bpm cases, TKE emerged by mid systole and dissipated by mid diastole, whereas at 137 bpm it persisted into mid diastole before decaying by end diastole. Average TKE at mid systole/peak systole/mid diastole for the MCA aneurysm was 4.36/4.39/0.99, 4.18/5.09/2.09, and 7.59/9.49/6.04 (all values x10^-5^), while for the LVA aneurysm average TKE was 3.42/5.98/0.90, 8.70/7.14/2.70, 8.68/14.4/6.78 (all values x10^-5^) for the 60 bpm, 100 bpm, and 137 bpm cases, respectively. Overall, heart rate drove significant increases in the magnitude and spatial footprint of TKE in both the MCA and LVA aneurysms.

**Fig 7:**
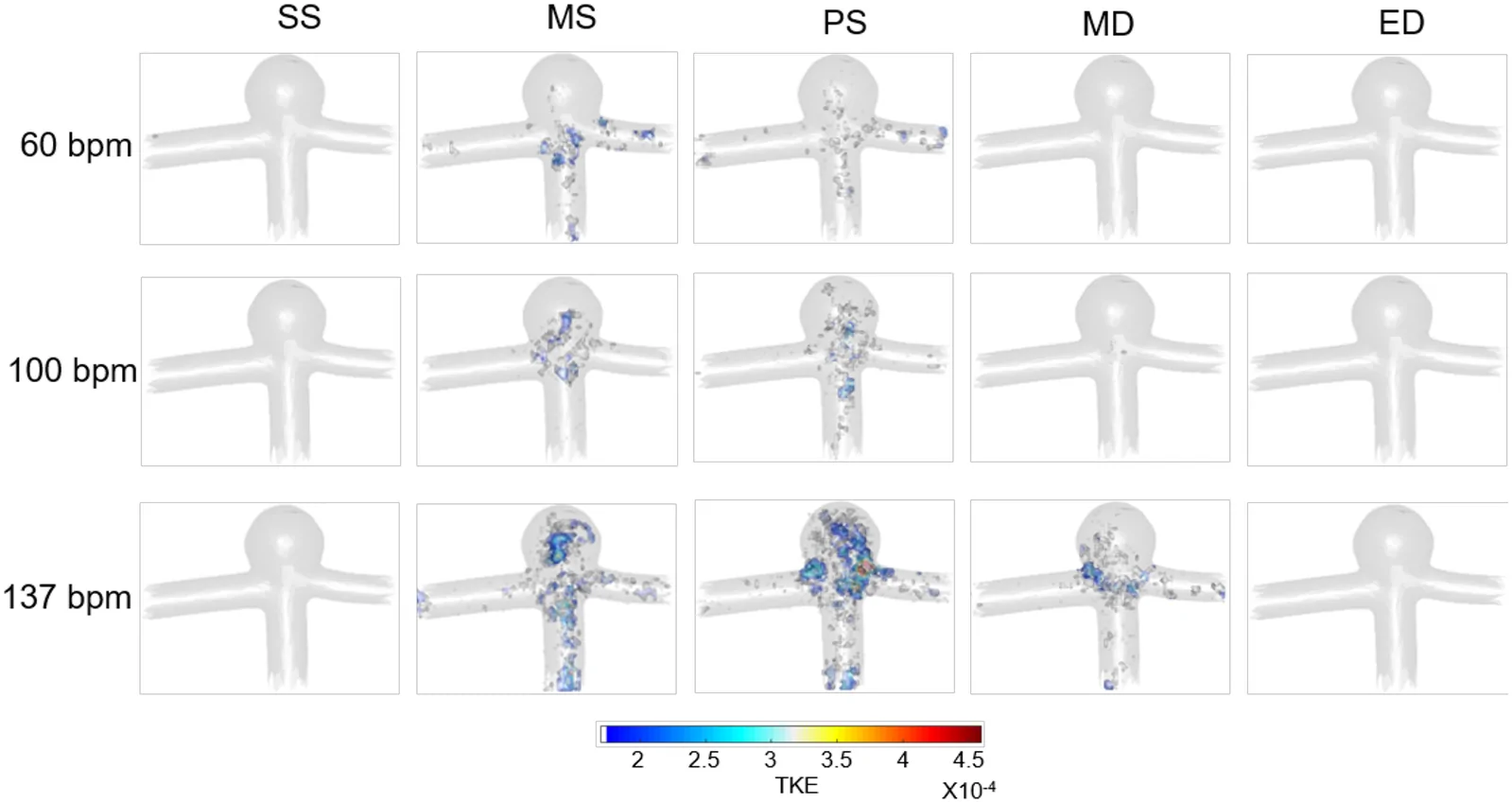
3D TKE for the MCA at start systole (SS), mid systole (MS), peak systole (PS), mid diastole (MD), end diastole (ED) for the 60 bpm, 100 bpm, and 137 bpm cases.

**Fig 8:**
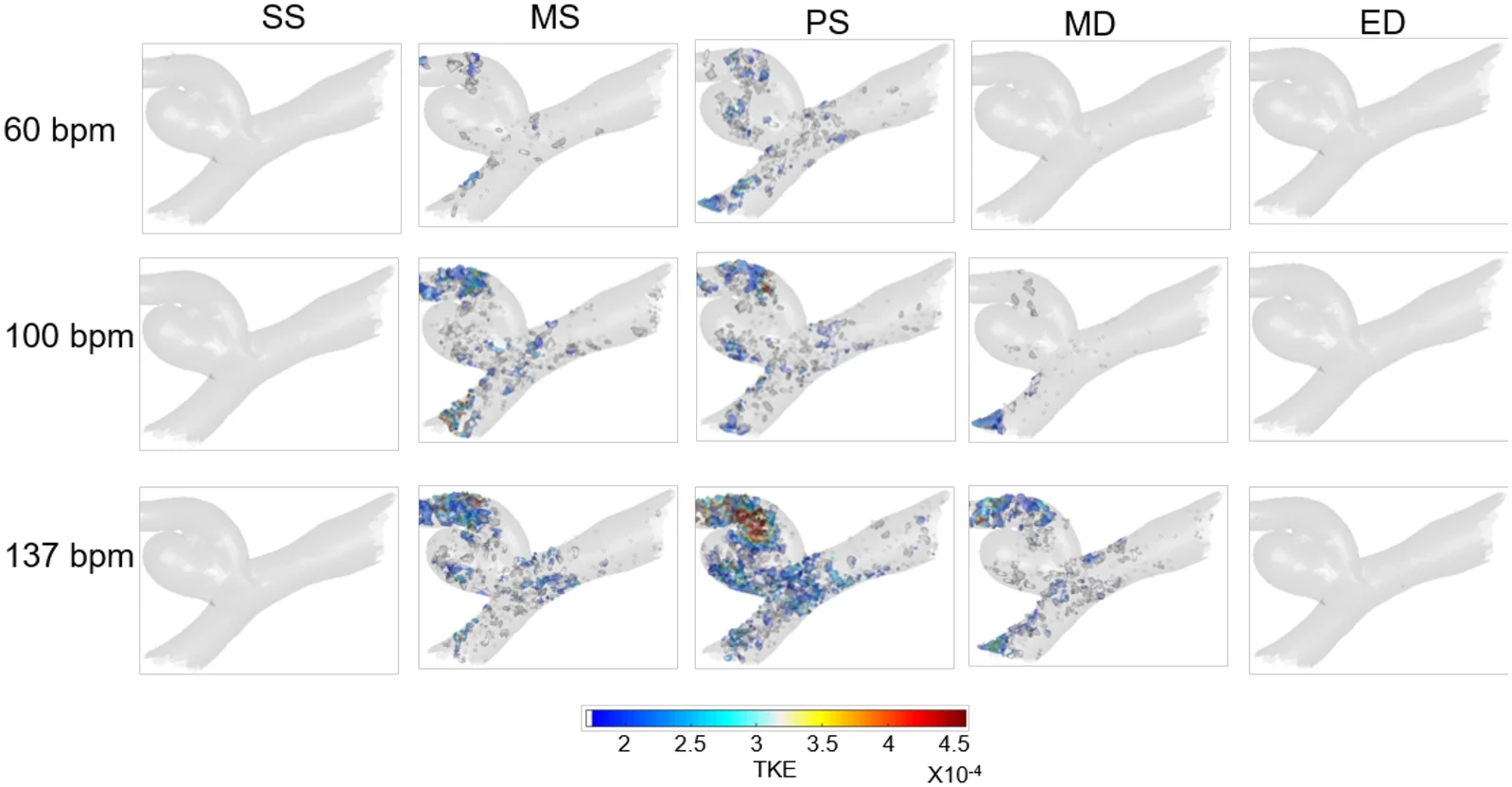
3D TKE for the LVA at start systole (SS), mid systole (MS), peak systole (PS), mid diastole (MD), end diastole (ED) for the 60 bpm, 100 bpm, and 137 bpm cases.

Fig 9 shows the WSS distributions normalized by the corresponding mean inlet WSS across the cardiac cycle and heart rates for the MCA aneurysm. At 60 bpm, high WSS regions occurred in the aneurysm neck and posterior/upper sac walls, which were extended and intensified as heart rate increased. Considering the pulsatile phase, a heart rate-dependent shift in timing of the maximum WSS was observed. For the 60 bpm case, WSS reached its maximum at peak systole, but this shifts to mid-diastole at 100 and mid-to-late diastole for the 137 bpm case. Normalized WSS inside the aneurysm sac at peak systole/mid diastole was 1.12/1.09, 1.37/1.83, and 1.55/1.80 for the 60 bpm, 100 bpm, and 137 bpm cases, respectively. In contrast, the LVA aneurysm (S2 Fig) maintained relatively heart rate invariant behavior, with similarly sized high WSS regions localized to the bifurcation area and IA sac near the posterior outlet observed in all cases. Normalized WSS inside the LVA aneurysm sac at peak systole/mid diastole was 0.99/0.83, 0.99/0.86, 1.08/1.00 for the 60 bpm, 100 bpm, and 137 bpm cases, respectively. Hence, peak systole WSS varied by only about 8% across cases, while mid diastole WSS was within 17% for all cases. Thus, this analysis indicates geometry-dependent effects of heart rate amplifies on WSS.

**Fig 9:**
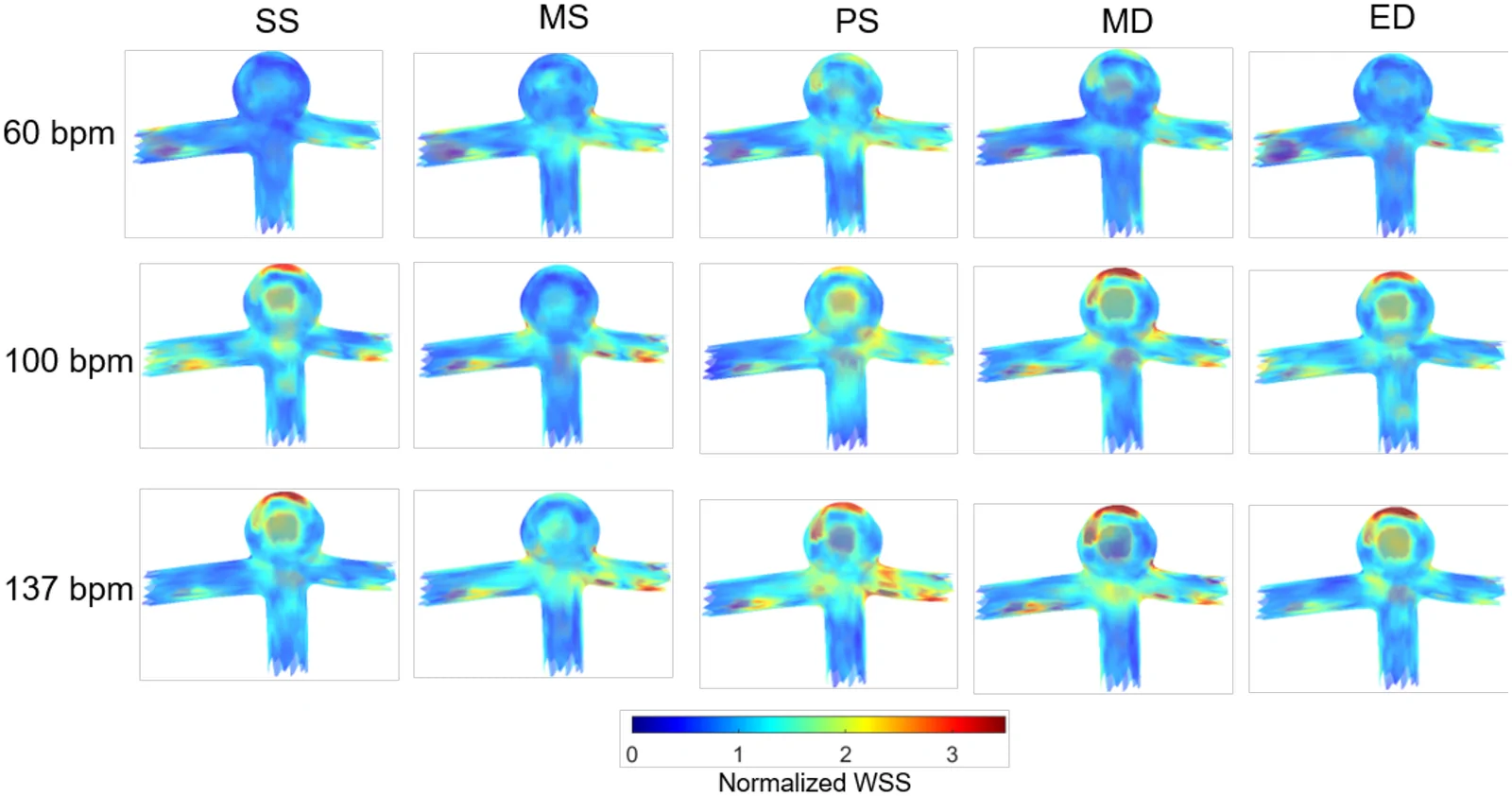
3D WSS normalized by inlet WSS for the MCA at start systole (SS), mid systole (MS), peak systole (PS), mid diastole (MD), end diastole (ED) for the 60 bpm, 100 bpm, and 137 bpm cases.

Fig 10 shows the OSI distributions for both geometries across heart rates. In both geometries, OSI maintained relatively different distributions for each heart rate case. Elevated OSI generally co-located with low WSS regions. In the MCA aneurysm, some similarities between the 60 bpm and 137 bpm OSI were observed (i.e., a high OSI region along the anterior sac wall), but generally no distinct trend of how OSI changes with heart rate was observed. Average OSI magnitude in the MCA aneurysm was 0.122, 0.104, and 0.120 for the 60 bpm, 100 bpm, and 137 bpm cases, respectively. In the LVA aneurysm, average OSI magnitude inside the aneurysm dome was 0.076, 0.096, and 0.100 for the 60 bpm, 100 bpm, and 137 bpm cases, respectively.

**Fig 10:**
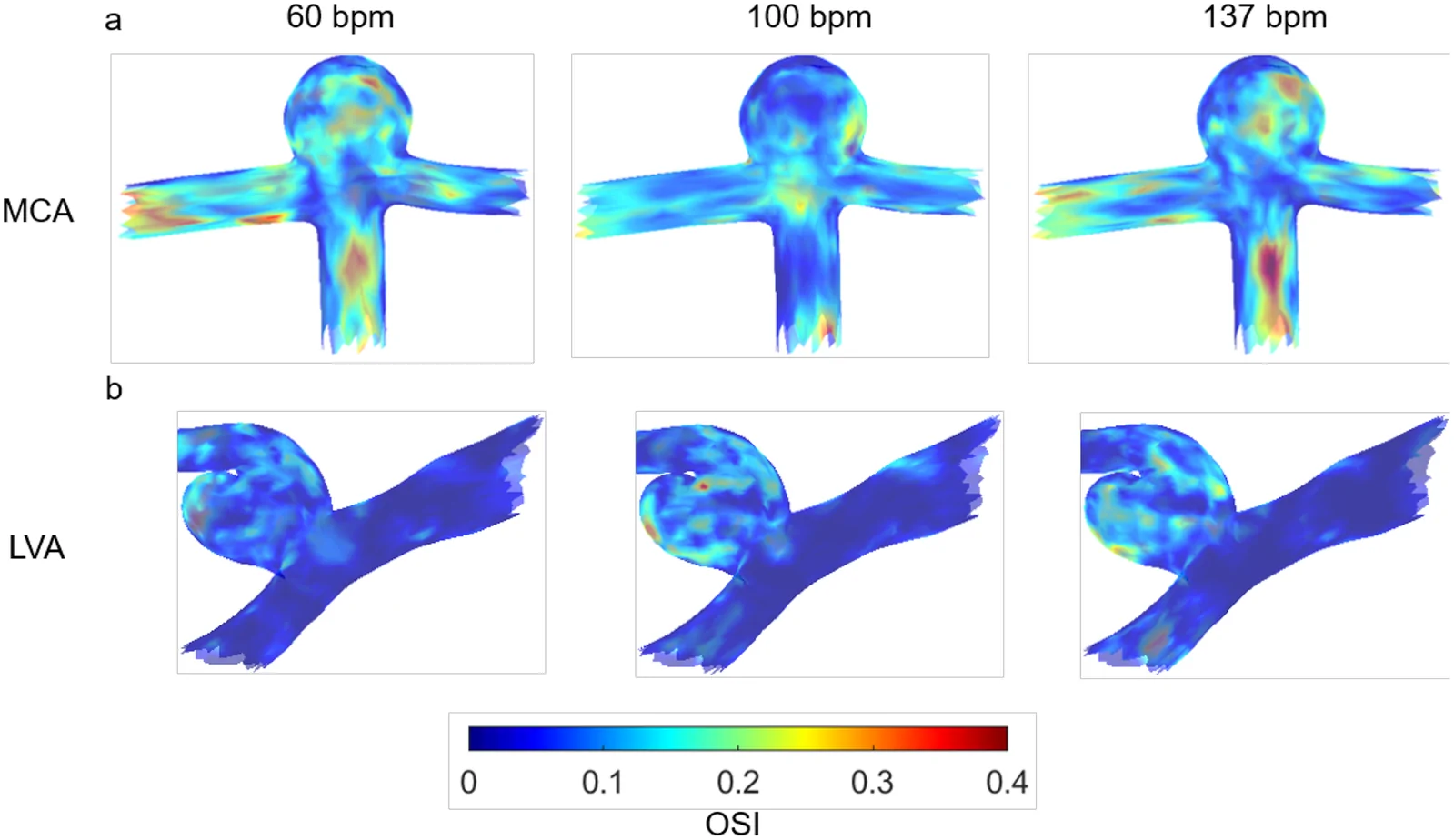
3D OSI for the MCA (a) and LVA (b) at start systole (SS), mid systole (MS), peak systole (PS), mid diastole (MD), end diastole (ED) for the 60 bpm, 100 bpm, and 137 bpm cases.

Fig 11 displays the normalized time averaged velocity (TAVEL) and normalized time averaged WSS (TAWSS) distributions across heart rates for the MCA (a and c) and LVA (b and d) aneurysms. For the MCA aneurysm, TAVEL had a slightly wider distribution at 60 bpm (IQR = 0.45, median = 0.50), whereas the 100 bpm (IQR = 0.40, median = 0.62) and 137 bpm (IQR = 0.39, median = 0.60) cases were similar. For the LVA aneurysm, TAVEL distribution for the 60 bpm case was also slightly broader (IQRs = 0.59, 0.55, 0.54) than the 100 bpm and 137 bpm, respectively. Additionally, median values slightly decreased from 60 bpm (0.81) to 137 bpm (0.73). In the MCA aneurysm, TAWSS had similar distributions with medians of 1.00, 1.10, and 1.15; and IQRs of 0.62, 0.65, and 0.64 for the 60 bpm, 100 bpm, and 137 bpm cases, respectively. For the LVA aneurysm, TAWSS distributions also remained similar across heart rates (IQRs ~ 0.71–0.77), with medians around 1.12. Overall, normalized TAVEL and TAWSS effectively reduced inflow bias, providing comparable hemodynamic insights across heart rates.

**Fig 11:**
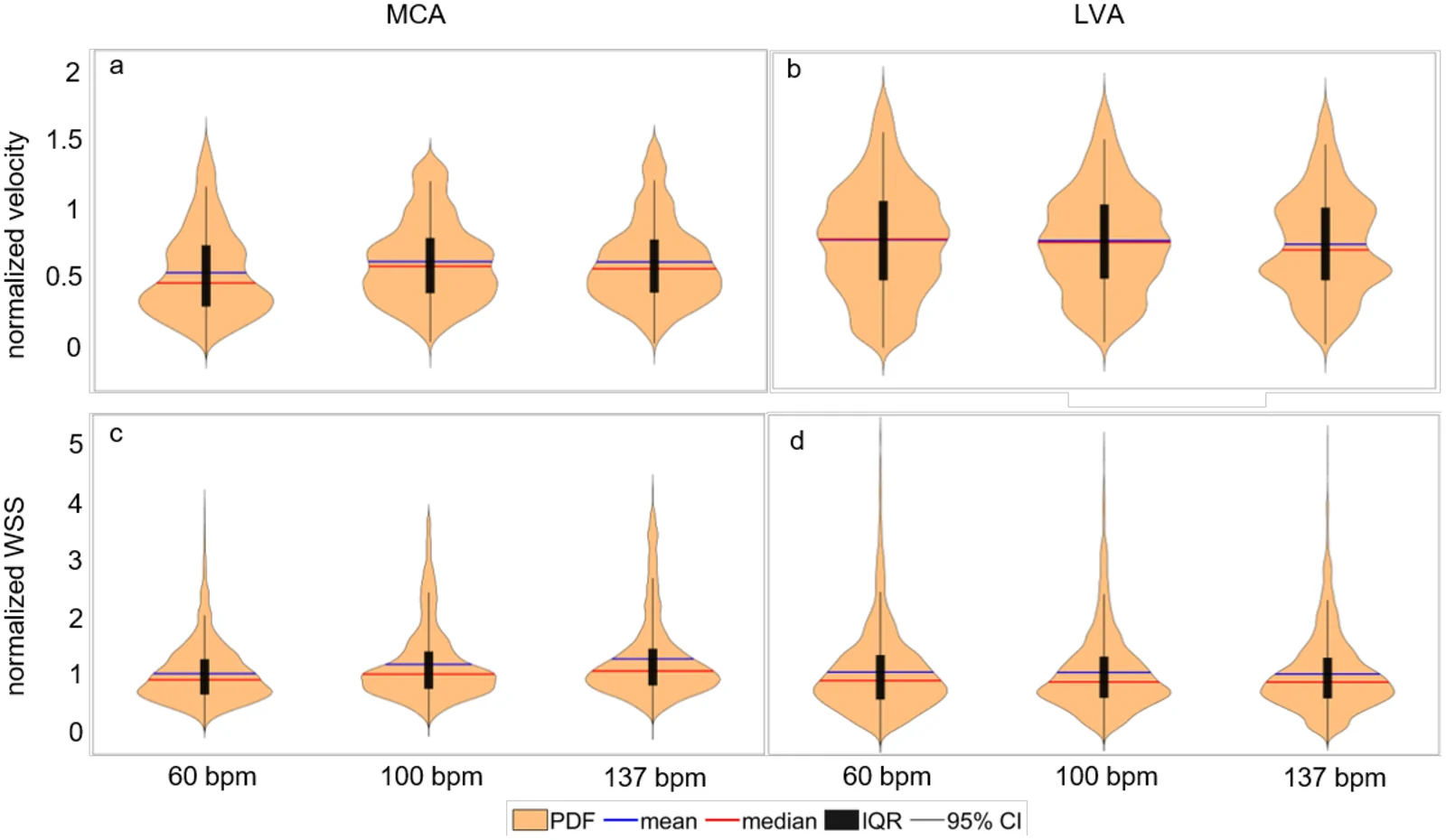
Distributions of Vel mag and TAWSS for (a and b) MCA and (c and d) LVA at all heart rates.

## 4. Discussion

In this work, we investigated how physiological factors like heart rate and inflow waveform amplitude, which can be constantly varying *in vivo*, affect IA hemodynamic parameters that have been associated with IA growth and rupture. We used volumetric PTV to study two patient-specific intracranial aneurysms (MCA and LVA aneurysms) at three heart rates that simulate rest, active movement, and exercise (or emotional stress) conditions. We analyzed the specific relationships between heart rate and flow hemodynamics, namely velocity, coherent structures, TKE, WSS, and OSI.

Increasing heart rate did not simply amplify peak systolic inflow; it altered the temporal character of the intra-aneurysmal flow. In the MCA aneurysm, the 100 bpm and 137 bpm cases produced a stronger inflow jet and a more compact vortex that remained anchored in the sac through mid- and end-diastole (and, at 137 bpm, into the onset of the next systolic phase). At 60 bpm, by contrast, the vortex weakened by end-diastole and its core migrated over a larger distance in the sac. Taken together, these observations indicate that higher heart rates promote not only stronger but also more spatially and temporally coherent rotational flow inside the aneurysm. These differences were consistent with the coherent-structure and TKE fields. For both aneurysm geometries, increasing heart rate increased the magnitude, spatial extent, and lifetime of coherent structures, and similarly elevated TKE intensity, coverage, and persistence past peak systole, with a marked escalation at 137 bpm. Hence, elevated heart rate injected more kinetic energy into the aneurysm and sustained that energy over a longer fraction of the cycle instead of allowing the flow to return to a quiescent, weakly circulating state during diastole. Importantly, this persistence beyond peak systole means that disturbed and vortical flow was no longer confined to the high-flow portion of the waveform (i.e., peak systole). This implies that tachycardic conditions may extend the duration of hemodynamic loading on the aneurysm wall, not just increase its peak magnitude.

The two aneurysm geometries responded markedly differently to the same imposed heart rate-dependent inflow conditions, supporting a strong role of patient-specific geometry in determining the hemodynamic response. Because identical inflow waveforms were imposed in both models and their inlet diameters were similar (3.45 mm for the MCA and 3.50 mm for the LVA), the resulting inlet Reynolds and Womersley numbers differed by only approximately 1–2% between geometries at a given heart rate. Thus, the substantial differences observed in intra-aneurysmal flow organization and wall loading cannot be explained by meaningful differences in the imposed inlet waveform or dimensionless inlet conditions between the two models. The MCA aneurysm behaved as a highly sensitive system: increasing heart rate from 60 bpm to 100 bpm and 137 bpm produced a stronger, more focused inflow jet, a tighter and more persistent intra-saccular vortex, higher WSS magnitudes and peaks, and broader spatial exposure to elevated WSS. These changes were accompanied by stronger coherent structures and elevated TKE that persisted into late diastole. In contrast, the LVA aneurysm showed a more muted response: streamline topology changed minimally across heart rates, WSS varied only slightly in magnitude and coverage, and although coherent structures and TKE did increase with heart rate, their intensity, spatial extent, and lifetime were consistently lower than in the MCA aneurysm. Together, these findings suggest that some aneurysms act as hemodynamic amplifiers of heart rate (MCA aneurysm), where increases in heart rate drive relatively larger intra-saccular rotation, shear, and mixing. Others (LVA aneurysm) behave more like low-pass filters. Differences in aneurysm morphology might explain this. The MCA has a larger aneurysmal sac and the inflow jet is primarily directed into the IA sac. On the other hand, the inferior outlet in the LVA aneurysm provides a low resistance outflow pathway through which excess momentum can be directed, such that even at high heart rates, the sac does not develop a dramatically stronger or more persistent vortex, and wall loading remains comparatively stable. This would suggest that the magnitude of heart rate effects on aneurysm hemodynamics is more pronounced when inflow-to-outflow vessel angles are greater which requires heart rate-induced momentum and energy increases to be directed through the aneurysmal sac.

Changes in heart rate also altered the wall loading environment. In the MCA aneurysm, higher heart rates produced higher WSS magnitudes, higher local peaks, and broader spatial coverage of elevated WSS. Importantly, at 100 bpm and 137 bpm the maximum intra-saccular WSS did not occur only near peak systole; elevated WSS persisted into mid diastole and remained high through end diastole. This means that at higher heart rates the wall experiences sustained high shear over a larger fraction of the cardiac cycle, rather than a brief systolic spike followed by recovery. In contrast, the LVA aneurysm showed only minor changes in WSS magnitude and distribution with heart rate, consistent with its weaker overall sensitivity. Across both geometries and all heart rates, regions of elevated OSI were consistently co-located with zones of low to moderate WSS rather than with the highest-shear regions. This co-location implies that increasing heart rate does two things simultaneously: it expands and prolongs areas of high instantaneous shear load, and at the same time it preserves pockets of slow, oscillatory near-wall flow elsewhere in the sac. In other words, rather than pushing the aneurysm wall toward a single uniform stress state, higher heart rates create a more heterogeneous shear landscape with some regions experiencing sustained high shear late into diastole, and other regions experiencing low-magnitude but highly oscillatory shear.

Oscillatory shear index (OSI) provided complementary information about how the directionality of near-wall shear evolved with heart rate. In the MCA aneurysm, the 100 bpm case maintained the lowest OSI values inside the sac, whereas the 60 bpm and 137 bpm cases exhibited higher OSI. One interpretation is that at 100 bpm the stronger, more anchored recirculation imposes a more consistent shear direction on the sac wall, reducing shear reversal even as absolute WSS increases. At 60 bpm, the weaker vortex drifted over a larger distance and decayed by end-diastole, which may promote more frequent changes in shear direction. At 137 bpm, the flow becomes more strongly disturbed, with elevated TKE and persistent vortical motion extending across the cycle, which can again reintroduce directional fluctuations. Taken together, the three heart rates correspond to three distinct hemodynamic regimes. At low heart rate (60 bpm), the sac experiences weaker shear but more variable shear direction. At intermediate heart rate (100 bpm), the sac experiences stronger and more temporally sustained shear, but with reduced oscillatory character at the wall. At high heart rate (137 bpm), the system transitions toward a persistently energized, more strongly disturbed state characterized by elevated TKE, longer-lived coherent structures, and high shear that carries into late diastole and even the next cycle. This non-monotonic structure implies that, under certain conditions, it is feasible for an elevated heart rate to produce a more advantageous hemodynamic environment (i.e., less likely to promote aneurysm progression). Moreover, this may imply one mechanism by which mild to moderate exercise could promote aneurysm health, but this notion should be more explicitly considered in future work.

There were several limitations of this study. The data for the 60 bpm and 100 bpm cases were collected in the same session while the 137 bpm case used here was collected at a different data collection session. This might introduce some uncertainties due to small differences in calibration error. The calibration error for the 137 bpm case was 0.00724 while the calibration error for the other cases was 0.00605, a difference well within tolerance for an experimental analysis. An additional limitation is that cardiac frequency and inflow magnitude were varied concurrently to reproduce physiologically motivated heart-rate conditions. Consequently, Reynolds and Womersley numbers both increased with heart rate, and the present experiments cannot uniquely separate the independent contributions of pulsation frequency and inertia to the observed hemodynamic changes. Nevertheless, the tested Reynolds number range remained within the same overall bulk flow-regime range across conditions, with no abrupt transition between distinct flow regimes. The reported results should therefore be interpreted as the integrated hemodynamic response to the coupled changes in cardiac frequency and inflow magnitude associated with the modeled physiological states, rather than as the isolated effect of pulsation frequency alone. Also, the MCA aneurysm model was flipped upside down and the inflow tube connected to the inlet was slightly tilted due to experimental limitations. Such conditions can introduce uncertainties when comparing values in this work to other studies for the purpose of establishing a universal trend. However, these conditions are not expected to have had any effect on the validity of the trends and relationships reported in this work. Another limitation of this study is the insufficient resolution of flow measurements at the posterior outlet of the LVA aneurysm, owing to experimental and hardware constraints. Furthermore, the inflow waveforms applied to the LVA model were intentionally matched to those imposed in the MCA model to enable a controlled comparison of the response of the two patient specific geometries under equivalent inlet forcing. Because the MCA and LVA inlet diameters were similar, this also resulted in closely matched Reynolds and Womersley numbers between the models at each heart rate condition. However, these imposed waveforms should not be interpreted as representative of native LVA physiology. Consequently, the cross-model comparison is intended to characterize geometry-dependent responses under controlled inflow conditions rather than differences between the native physiological hemodynamics of the MCA and vertebral artery territories. Another limitation was sample size, as only six cases were investigated (three heart rates for two IAs). Future work should prioritize expanding the sample size and the acquisition of region-specific inflow waveforms. Another limitation is the effectively rigid nature of the block-cast PDMS phantoms. Although the block-cast construction substantially limits wall deformation compared with thin-walled compliant vascular phantoms, model compliance was not directly quantified. In a more compliant model, pulsatile pressure loading could produce cyclic deformation of the parent vessel, aneurysm neck, and sac, thereby altering the instantaneous flow field and wall loading parameters and potentially modifying the observed sensitivity to heart rate. The magnitude and direction of these effects would depend on the local geometry, wall mechanical properties, and pressure loading. Thus, the present results should be interpreted as characterizing effectively rigid wall hemodynamics, and future studies using compliant phantoms would be valuable for assessing fluid–structure interaction effects.

## 5. Conclusions

In this work, we used volumetric PTV to characterize how heart rate modulates intra-aneurysmal hemodynamics in two patient-specific intracranial aneurysms across three physiologically motivated heart rates (rest, moderate exertion, and high exertion/stress). We found that increasing heart rate did not simply increase instantaneous flow magnitude; it changed the timing and persistence of disturbed flow. In the MCA aneurysm, higher heart rates generated a stronger inflow jet, an anchored recirculating vortex, and elevated coherent-structure strength and TKE that persisted into mid- and end-diastole, preventing the sac from returning to a quiescent state between beats. This also broadened the spatial footprint and duration of elevated wall shear stress and produced sustained shear loading later in the cycle. By contrast, the LVA aneurysm showed comparatively muted changes in streamline topology, WSS, and OSI with heart rate. Together, these results suggest that some aneurysms act as hemodynamic amplifiers of heart rate, while others behave more like buffers, so the hemodynamic impact of tachycardia is morphology-dependent rather than universal. Finally, the MCA aneurysm exhibited non-monotonic behavior: the intermediate heart rate corresponded to a distinct regime of sustained but more directionally consistent shear, indicating that a higher heart rate can yield negative or positive hemodynamic effects, depending on specific conditions.

## Supporting information

S1 Fig 3D time averaged velocity streamlines normalized by inlet velocity for the LVA at 60 bpm, 100 bpm, 137 bpm cases. (PDF)

S2 Fig 3D WSS normalized by inlet WSS for the LVA at start systole (SS), mid systole (MS), peak systole (PS), mid diastole (MD), end diastole (ED) for the 60 bpm, 100 bpm, 137 bpm cases. (PDF)
