# Supplementary material for: Effect of heart rate on hemodynamics in patient-specific intracranial aneurysms: An in vitro study
Source: PLoS One. 2026 Oct 29;21(10):e0359941. doi: 10.1371/journal.pone.0359941 (PMC13718917; doi:10.1371/journal.pone.0359941)
Supplement: Supplementary file 1 [file pone.0359941.s001.pdf]

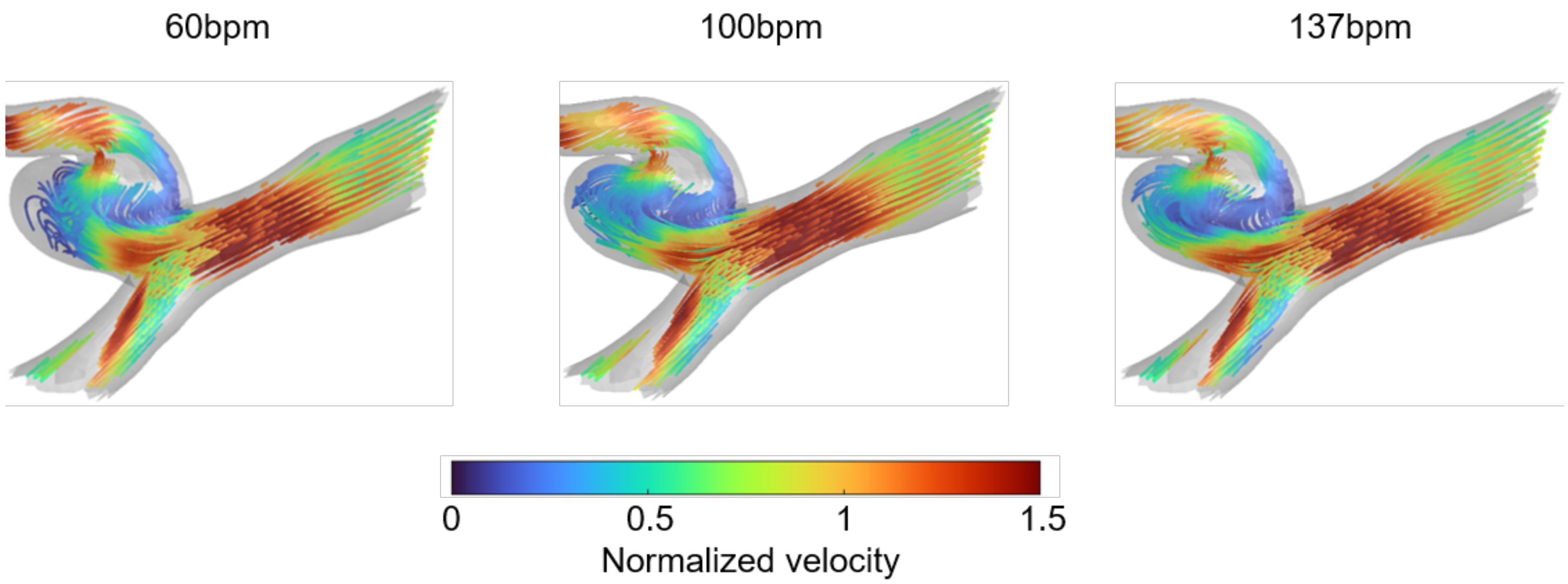
60bpm
100bpm
137bpm
0
0.5
1
1.5
Normalized velocity
