# Supplementary material for: Effect of heart rate on hemodynamics in patient-specific intracranial aneurysms: An in vitro study
Source: PLoS One. 2026 Oct 29;21(10):e0359941. doi: 10.1371/journal.pone.0359941 (PMC13718917; doi:10.1371/journal.pone.0359941)
Supplement: Supplementary file 2 [file pone.0359941.s002.pdf]

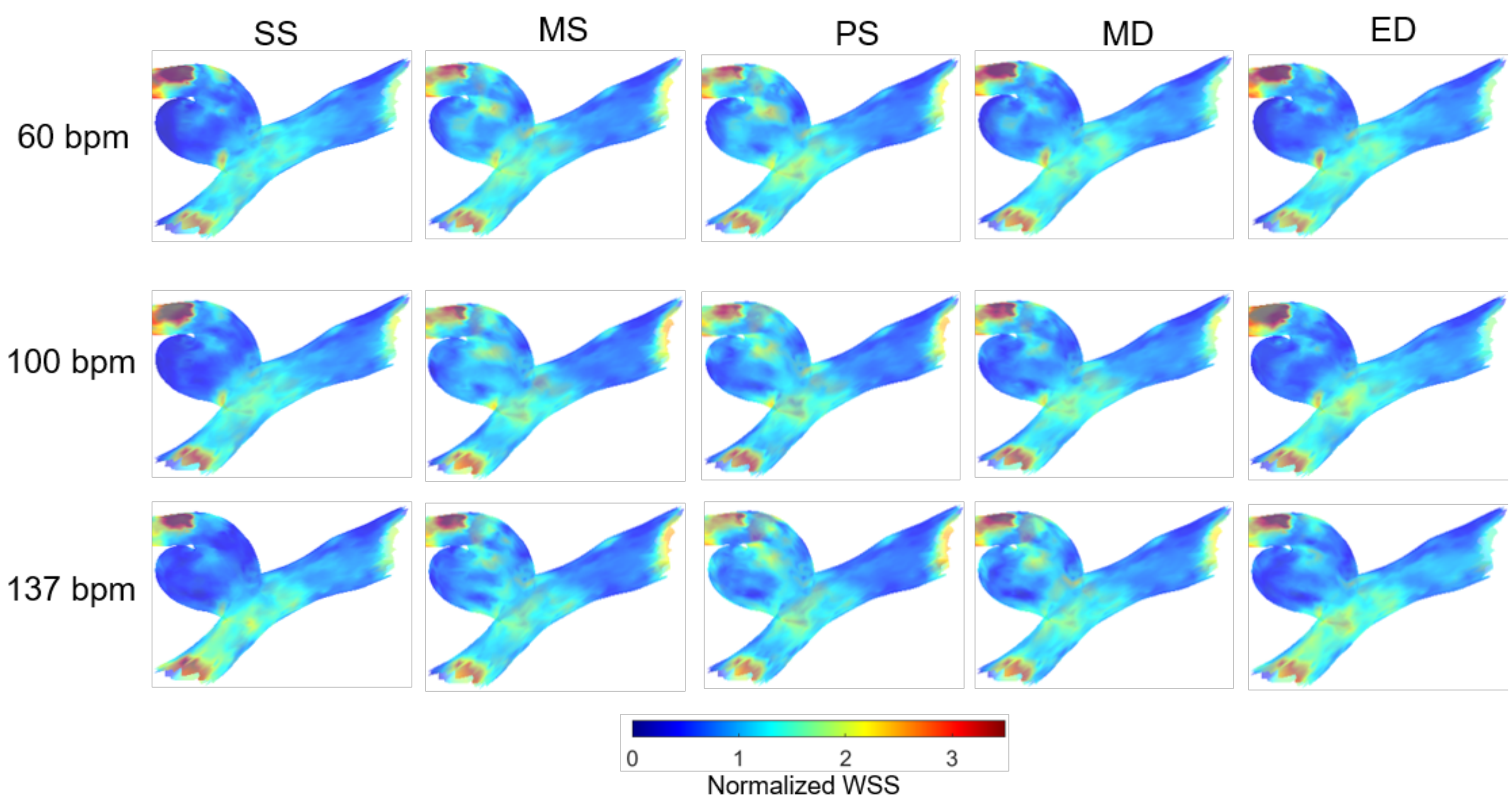
SS
MS
PS
MD
ED
60 bpm
100 bpm
137 bpm
0
1
2
3
Normalized WSS
